# Chromatographic Methods Developed for the Quantification of Quercetin Extracted from Natural Sources: Systematic Review of Published Studies from 2018 to 2022

**DOI:** 10.3390/molecules28237714

**Published:** 2023-11-22

**Authors:** Daniel Carvalho, Cláudia Pinho, Rita Oliveira, Fernando Moreira, Ana Isabel Oliveira

**Affiliations:** 1 Escola Superior de Saúde, Instituto Politécnico do Porto, Rua Dr. António Bernardino de Almeida, 4200-072 Porto, Portugal; 10170218@ess.ipp.pt (D.C.); clp@ess.ipp.pt (C.P.); rfo@ess.ipp.pt (R.O.); aio@ess.ipp.pt (A.I.O.); 2 Centro de Investigação em Saúde e Ambiente (CISA), Escola Superior de Saúde, Instituto Politécnico do Porto, Rua Dr. António Bernardino de Almeida, 4200-072 Porto, Portugal; 3 REQUIMTE-LAQV, Escola Superior de Saúde, Instituto Politécnico do Porto, Rua Dr. António Bernardino de Almeida, 4200-072 Porto, Portugal

**Keywords:** analytical methods, chromatography, flavonoids, quercetin, systematic review

## Abstract

Quercetin (QUE) is the most widely used flavonoid for therapeutic purposes. To improve the available knowledge about the properties of some natural products, determining the amount of QUE is crucial. The main objective of this systematic review is to identify the analytical methods validated for detecting and quantifying QUE in different matrices and characterize their sensitivity. A search was conducted until 30 June 2023 in the PubMed database for experimental studies that addressed the validation of chromatographic analytical methods to detect and quantify QUE from consumable natural products. Only studies published between 2018 and 2022, written in English, were included. The risk of bias was assessed by emphasizing methods of comparison according to previously published studies. Descriptive statistics were used to depict the obtained results. The studies were analyzed based on the type of QUE source, chromatographic method, and validation parameters. A total of 17 studies were included in this review. Plants were the most commonly analyzed source of QUE. Among the detection methods, spectrophotometry proved to be the most widely used, surpassing mass spectrometry (MS). After analyzing the bias, all the included studies mentioned/presented, totally or partially, at least four of the eight parameters.

## 1. Introduction

Flavonoids are antioxidant compounds commonly found in vegetal origin products that show multiple potentialities in human health, substantiated by their antiallergic, antiviral, anticancer, anti-inflammatory, and cardiovascular documented activities [1,2]. This class of secondary metabolites belongs to a group named phenolic compounds, where lignans, tannins, phenolic acids, and stilbenes are also included but where flavonoids remain the major compounds, being widely spread throughout the plant kingdom and found in several fruits and vegetables [3,4,5,6,7,8]. Flavonoids are structurally composed of two benzene (A and B) rings linked to a heterocyclic (C) ring (two aromatic and one oxygenated ring) with a 15-carbon structure (C6-C3-C6). Flavonoids are often found in nature as aglycones but also conjugated with organic acids or sugars [3,5,6,7,8,9,10].

According to their chemical structure, more specifically with the degree of hydroxylation of the central ring, flavonoids can be divided into different subclasses (flavonols, isoflavones, flavones, anthocyanins, flavanones, and flavan-3-ols) [3,5,7,8,9,11].

QUE is a flavonoid with multiple potentialities in human health. It is the most widely used flavonoid in the treatment of various diseases, which may be related to its properties, such as antioxidant, anti-inflammatory, and prevention of cardiovascular and neurodegenerative diseases. This flavonol is present in multiple vegetal sources, namely plants, fruits, and vegetables (e.g., onions, apples, tea, brassicas, grapes, nuts, *Hypericum perforatum*, *Ginkgo biloba*, *Sambucus canadensis*, and *Aesculus hippocastanum*) [1,2,12,13,14].

Chromatography is an analytical technique that has been one of the most widely used techniques for analyzing compounds or mixtures of compounds, such as products of plant origin, to identify their chemical composition and determine the number of compounds present in each sample [15,16,17,18].

Determining the amount of QUE present in natural sources is an important step towards improving knowledge about the properties related to plant sources, as well as, in the case of therapy, being able to determine the amount of compound to be administered and quantify the amount of QUE present in new formulations.

The present review mainly aimed at identifying the analytical methods developed and validated for detecting and quantifying QUE in different matrices from vegetables, fruits, medicinal plants, and other natural consumable products. Additionally, it aimed at featuring the sensitivity of the developed methods to clarify the minimum required concentration of QUE usually detected. Given the vast number of studies presenting alternatives to detect QUE, if someone pretends to adopt a methodology previously published, choosing the most appropriate method (particularly regarding column choice and mobile phase definition) might be very challenging. By featuring the publicly available analytical methods, it was possible to evaluate and compare their accuracy and precision, but especially their sensitivity. This might be a pivotal criterion when choosing a methodology according to the expected concentration of QUE in the analyzed matrix. This review provides an overview of validated methods, facilitating the adaptation of the most convenient method for analyzing QUE content, depending on the existing conditions.

## 2. Materials and Methods

### 2.1. Database Search/Search Strategy

For this review, a search in the PubMed database was performed, considering studies published between 2018 and 2022, using the following search terms: “quercetin” AND (detection OR analysis OR determination OR assessment OR identification OR quantification) AND (HPLC OR high-performance liquid chromatography OR GC OR gas chromatography OR UHPLC OR UPLC). The last search date was 30 June 2023.

### 2.2. Article Analysis

All retrieved results from the database search were read regarding title and abstract. Studies with high relevance to the theme (e.g., an abstract suggested that a chromatographic method was applied to detect and/or quantify QUE from a plant-related matrix) were fully read. After applying the complete eligibility criteria, the studies were included or excluded.

Only experimental studies published in English between 2018 and 2022 that addressed the development and validation of analytical methods by chromatography to detect and quantify QUE from consumable natural products were included in this review. Studies were excluded if they: (i) consisted of literature reviews; (ii) only addressed compounds derived from QUE but not the compound itself; (iii) did not present the main characteristics of the analytical method (e.g., retention time, chromatographic technique and detection equipment); (iv) did not identify the specific natural product in which the analytical method was used (e.g., mixtures were excluded); (v) did not present the results of the validation of the analytical method, at least for the following main parameters: sensitivity (limit of detection (LOD) and/or limit of quantification (LOQ)), precision and accuracy; and (vi) pharmacokinetic studies.

All results were analyzed by two independent researchers, and when there was no consensus as to include or exclude a specific study, a third researcher decided.

Collected results were grouped regarding separation technique and detection method and the nature of the matrix (plant material—fruit, leaves, root, dry grass, stems, rhizomes, rind, flowers, pod, cladodes, and seeds—or seaweed).

### 2.3. Information Collection

A systematization of the results to be collected from each study was elaborated, resulting in a summary table of results. These data were collected by two researchers independently, and inconsistencies were subsequently jointly analyzed and discussed.

Collected results consisted of (i) separation technique and detection method, (ii) mobile phase and stationary phase characteristics, (iii) analysis length, (iv) LOD and/or LOQ, (v) precision, and (vi) accuracy. Other data concerning the mobile phase and stationary phase of the chromatography technique were collected, and the identification of all analytes targeted by each study was also sought out.

The real amount of QUE was tabulated in specific groups and synthesized according to their source: fruits, vegetables, medicinal plants, and others.

To facilitate the comparison between studies, the units of mass were unified in all studies despite their original presentation. Regarding sensitivity measurement, LOD or LOQ were calculated using presented data regarding the other unit (e.g., if LOQ was absent, but LOD was present, LOQ resulted from LOD × 3; if LOD was absent, but LOQ was present, LOD resulted from LOQ/3) [19].

As to precision determination, if the presented value resulted from the relative standard deviation (RSD) (%), precision conversion data resulted from 100 (%)—RSD (%).

### 2.4. Assessment of the Risk of Bias

To assess the risk of bias, emphasis was placed on comparing methods according to previously published studies [20]. Accordingly, the assessment did not serve to exclude studies but rather to provide an overall characterization of the developed methods and to compare them regarding their efforts to limit bias (e.g., the average deviation from a true value). The considered variables were (i) previous establishment of criteria of acceptable performance; (ii) comparison of test method with reference method using reference material; (iii) presentation of x–y plot of data with eye examination; (iv) consideration of difference plot and statistics of difference; (v) consideration of regression analysis; (vi) performance of interference test; (vii) performance of linearity test; and (viii) performance of recovery test.

The mean difference was considered in the presentation of results to establish comparisons between different analytical methods regarding sensitivity measures.

### 2.5. Data Analysis

To depict the obtained results, descriptive statistics were used. Box and whisker plots were generated to compare LOD between different analytical methods owing to their visual effect and easy interpretation regarding percentiles, minimum, and maximum values. The prevalence of the use of each analytical method was also calculated to feature the present trends in analytical methodologies employed to detect QUE.

## 3. Results

**Figure 1:**
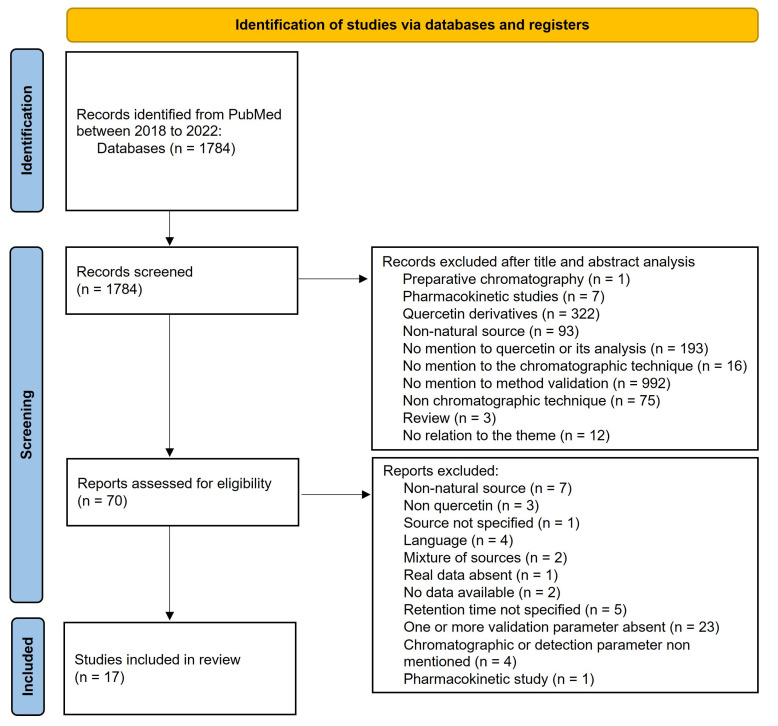
Methodology adopted for the selection of studies published in indexed journals and respective exclusion criteria.

In the present review, a total of 17 studies were included, depicting the validation of chromatographic methods to detect and quantify QUE from different matrices of natural consumable products (Figure 1).

Although some other studies presenting the chromatographic detection and quantification of QUE in different matrices were also published in recent years, those analyzing the flavonoid content in processed matrices, such as beverages, were excluded [21,22].

QUE is one of the most popular antioxidants and is widespread throughout the plant kingdom. Accordingly, the analysis of different vegetal sources to evaluate their QUE content has caught the attention of the scientific community, generally alongside other compounds with therapeutic potential.

### 3.1. Quercetin Sources

Table 1 summarizes the information collected from the publications included in this review regarding the analyte, plant source and sample, sample preparation and extraction procedure, and the amount of QUE in real samples.

As already mentioned, QUE is present in a variety of vegetal sources, so studying them became pertinent to understanding its properties better and possibly relating these properties to its chemical profile [40,41,42,43,44].

**Figure 2:**
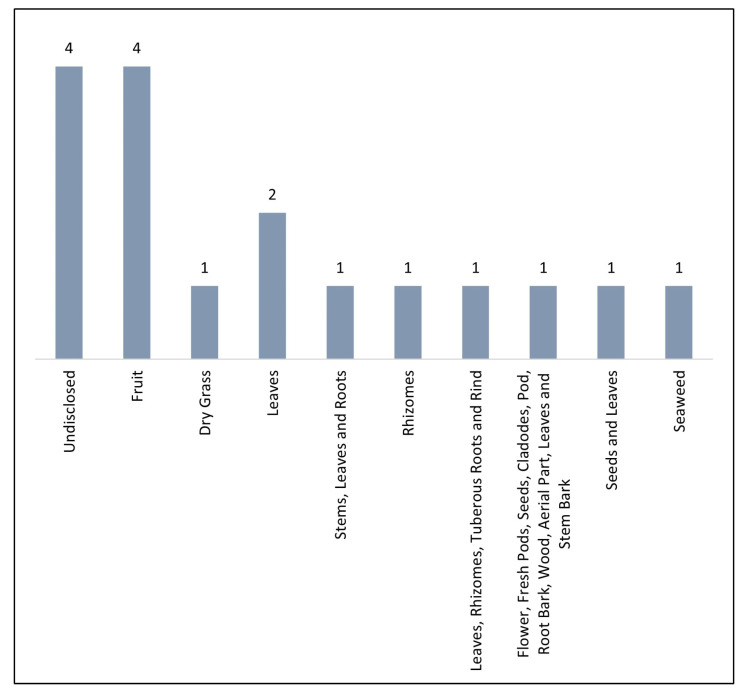
Number of original studies published between 2018 and 2022 that developed chromatographic methods to quantify quercetin, according to their sources.

Among plant sources, the most studied were plants regardless of the part of the plant used (12 of the articles) (Figure 2). This may be due to the potential of these and their traditional use for the prevention and treatment of various diseases, which has led to a growing need to understand what underlies their therapeutic effect and subsequently be able to use them correctly, with possible application in the pharmaceutical industry [40,41,42,43,44].

Among the plants cited in the studies included in this systematic review, a variety of origins, species, and biological activities is perceived, which is another indicator of the importance and availability of QUE. For example, *Diospyros khaki*, known as oriental persimmon, Japanese persimmon, and kaki, is mainly grown in China, Korea, Japan, Brazil, Italy, Israel, and New Zealand and is commonly used in Traditional Chinese Medicine to treat coronary heart disease and cerebral arteriosclerosis disease [26,45]. *Sambucus formosana* belongs to the genus Sambucus and is one of the various species called elderberry. This genus of plants is widely distributed throughout Europe, Asia, and North America. Specifically, *S. formosana* is a plant traditionally used as a blood circulation invigorating herb, and it is applied externally to treat trauma, infectious wounds, and inflammations by Taiwanese aborigines [29]. *Triplaris gardneriana* Wedd, also known as pajeu, can be found in the northeast of Brazil. It is used in traditional Brazilian medicine for the treatment of various diseases, such as bleeding, hemorrhoids, coughing, and bronchitis [37,46].

**Figure 3:**
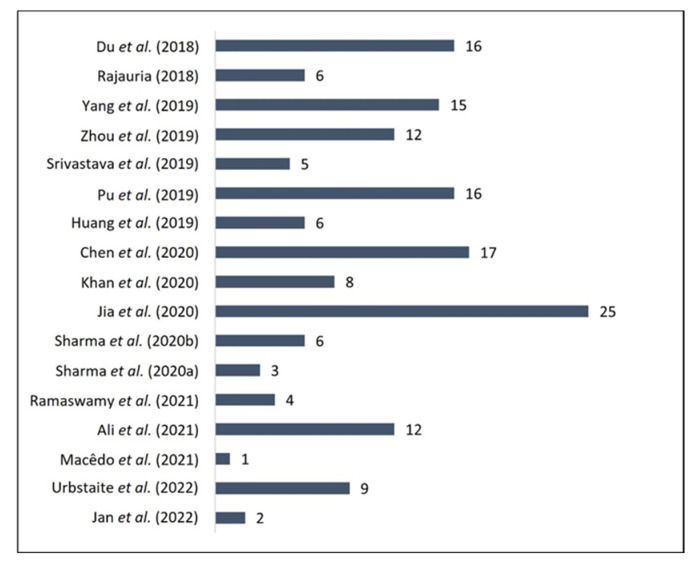
Number of analytes analyzed per study published between 2018 and 2022 for quercetin quantification through chromatographic methods [23,24,25,26,27,28,29,30,31,32,33,34,35,36,37,38,39].

Regarding the analytes, vegetal sources are complex matrices with an extensive phytochemical profile [44]. In this study, 16 of the included articles presented multi-analyte analyses; in other words, they performed detection or quantification, with an average of 10 analytes per included study (Figure 3). Only one study reported the detection of QUE alone [37]. The maximum number of analytes studied (25 analytes) was reported by Jia et al. [32].

Most articles analyzed between two and nine analytes (53%), followed by the articles that analyzed between 10 and 19 analytes (35%). The detection of one analyte or more than 20 was reported by 6% of the articles.

### 3.2. Sample Treatment Prior Chromatographic Analysis

Most of the included studies promoted the fragmentation of samples by pulverizing [23,27,28,29,33,37,38,39] or grinding [24,31,32]. This procedure increases the effectiveness of the extractive methods subsequently applied, namely by increasing the area of contact with extractive solvents. Different extractive methods were adopted to ensure the recovery and concentration of QUE, generally combining multiple methodologies amongst the most conventional (such as maceration [34], percolation [24,29,30,33,37,39], reflux extraction [25,26], and liquid–liquid extraction [25,26,29,30,33]) and/or the most recent (namely, ultrasound-assisted extraction [23,27,28,31,32,35,36,39], solid-phase extraction [24], and chromatography techniques for sample preparation [26,37]). The preference for three of these techniques was quite evident: percolation, liquid–liquid extraction, and ultrasound-assisted extraction. Briefly, during percolation, the plant materials are soaked with a selected solvent and left to stand in a well-closed container, after which the whole materials are covered with enough amount of the selected solvent for extraction [47]. This exhaustive process in which soluble constituents are removed by extracting the crude drug with fresh solvent was preferred over its main competitor, maceration, for QUE extraction. Although simpler and less expensive, maceration is also less effective [48]. As to other similar alternatives, reflux extraction was only used in two studies [25,26]. Reflux extraction is more efficient than percolation, requiring less extraction time and solvent, but cannot be used to extract thermolabile compounds, which might be relevant in multiple-analyte studies [49]. Still, in most studies using percolation, subsequent combination with other extractive procedure(s) was performed. As for liquid–liquid extraction, this technique is based on the partitioning of organic compounds between an immiscible organic solvent and the aqueous sample [50]. Finally, in ultrasound-assisted extraction, the ultrasounds passing through the samples create compression and expansion, ultimately forming cavitation and accelerating the dissolution of the solute and heat transfer, further improving extraction efficiency [47,49]. Since ultrasound-assisted extraction is best suited for solid plant samples, it is not surprising that all the methods described it for the extraction of QUE in solid samples, usually after pulverization or grinding [47]. Being an effective technique with low solvent and energy consumption that is applicable for the extraction of thermolabile and unstable compounds [49], ultrasound-assisted extraction was the most frequently mentioned extraction technique in the included studies.

Fourteen of the included studies also described the conduction of filtration prior to analysis, which might be crucial to eliminate matrix interference in the chromatogram and prevent equipment from deteriorating with larger particles [23,24,25,27,28,30,31,32,33,34,35,36,38,39].

### 3.3. Chromatographic Conditions

The information regarding chromatographic and detection conditions and validation parameters collected from the publications included in this review are summarized in Table 2. Table S1 summarizes the gradient programs employed in each study.

Liquid chromatography methods were preferred for QUE detection and quantification over gas chromatography (GC), being reported in 100% of the included studies. Although no study using GC had been identified that met the inclusion criteria for this review, the detection of QUE using GC is possible and has already been described in articles before the time range considered [51]. GC has several advantages (e.g., easy to apply, inexpensive, requires less solvent, allows the analysis of volatile compounds, and there is no interaction of the mobile phase with the analyte), and in the case of QUE, its high operating temperatures are not significantly destructive since QUE is one of the most thermally stable flavonoids [16,52,53]. However, as previously mentioned, all but one of the studies carried out multi-analyte analyses, including compounds that are less thermally stable than QUE and that could be destroyed in the GC analysis. In addition, GC generally involves laborious derivatization procedures that increase the likelihood of making a mistake in sample preparation. Previous studies that determined QUE by GC described derivatization procedures that may have discouraged more recent studies from using this technique [54,55].

**Figure 4:**
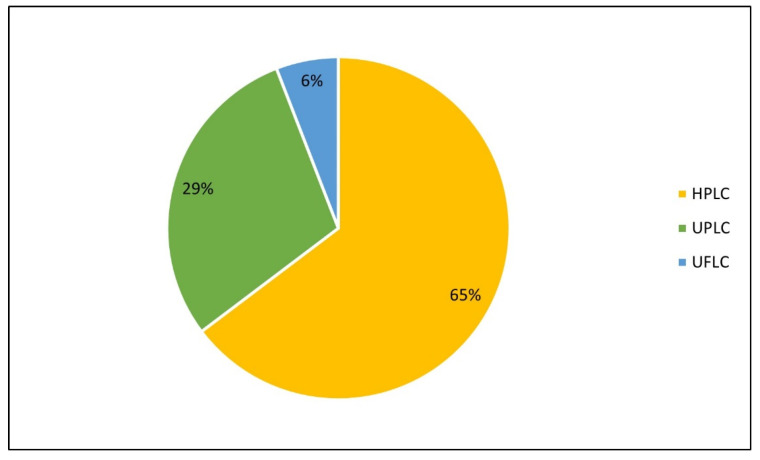
Percentage of original studies published between 2018 and 2022 that developed chromatographic methods to quantify quercetin, according to chromatographic methods. HPLC: high-performance liquid chromatography; UPLC: ultra-performance liquid chromatography; UFLC: ultra-fast liquid chromatography.

High-performance liquid chromatography (HPLC) was the most used liquid chromatographic technique for QUE analysis, being used in 65% of the studies (Figure 4).

Studies that were published in the years before the time range considered in this review had already stated that HPLC was the most widely used method for QUE detection [56,57,58,59].

Failing to present all the main characteristics of the analytical method deemed as relevant for data synthesis and/or not identifying the specific natural product in which the analytical method was used (mixtures of compounds were excluded), thin layer-chromatography (TLC) methods were not fully depicted in the context of this review. However, there are also recent TLC methods that determined QUE in Itrifal formulations of Unani medicine [60], polyherbal formulations containing Terminalia species [61] and *Myristica fragrans*, *Hemidesmus indicus*, and *Inula racemosa* herbs [34].

The mobile phase can be a single solvent or a mixture [16,52]. All the analyzed studies employed mobile phases composed of a mixture of solvents, and water was present in most of the described methods. In chromatographic methods using reverse-phase HPLC, such as those herein included, it is frequent to use a moderately polar aqueous mobile phase and a nonpolar stationary phase [62]. Since QUE is a polar compound, and in reverse-phase HPLC, there is a stronger attraction of the polar molecules to polar solvents than to the stationary phase, a faster elution is ensured when water-containing mobile phases are used [62,63]. It can also be seen that most of the studies use acidified water, which may be related to the advantages that acidification of the mobile phase brings, such as increased chromatographic resolution, allowing more defined peaks to be obtained, and better separation of the peaks of all the compounds present in complex mixtures, and possibly a reduction in the time needed for the chromatographic run [15,16,17,52,64,65].

Different types of acids can be included in the mobile phase for the chromatographic analysis of samples [66,67,68,69]. Formic acid was the most widely used chemical for acidifying the mobile phase (75%), followed by acetic acid (19%), making them the most widely used in recently developed chromatography methods for QUE quantification. Orthophosphoric acid was employed in 6% of the articles. These results are not surprising since formic acid (first) and acetic acid (second) are described as two of the most used acids in chromatography [70]. The importance of adding acidic solutions to the mobile phases for analyzing QUE is further emphasized by its chemical characteristics. Since QUE is a weak acid, it is degraded by hydrolysis in alkaline solutions and is, therefore, more stable in acidic conditions [71,72].

The organic solvent acetonitrile (ACN) was included in 76% of the described mobile phases, followed by methanol. This is a solvent with a high affinity for a great variety of compounds and which is capable of enhancing chromatographic resolution when used in higher proportions. However, this solvent is more expensive compared with methanol, therefore increasing the costs associated with the method. It is usually recommended to start with ACN when optimizing multi-analyte chromatographic methods, further increasing the probability of ACN being the chosen organic solvent [52,73,74]. ACN is also associated with a decrease in retention time due to its strong elution capacity [52].

Elution can occur in two modes: isocratic (constant proportion over the analysis time) or gradient (different proportions of each solvent over time), as the mobile phase may require adjustment over time depending on the polarity of the analyte, and the number of analytes present in the sample [16,52,69,75,76,77].

In the studies included in the present review, gradient mode elution stood out (88%) compared with the isocratic mode (12%). The preferential use of this mode for elution in the studies analyzed may be related to the complex mixtures injected, which correspond to high numbers of analytes, as previously evidenced.

Most studies use the isocratic mode due to its simplicity of application, but the gradient mode is widely used when the samples to be analyzed represent complex mixtures [69,77]. This trend can be seen in the studies presented in this review. In addition, the gradient mode has several advantages, such as the ability to increase the resolution of compounds with low retention and better elution of compounds with high retention, since it allows the affinity for the mobile phase to increase over time [75,76,77]. However, gradient mode should be avoided, as it might lead to a faster column degradation [76].

All the included studies used 18-carbon (C18) columns to analyze QUE. The so-called C18 columns or octadecylsilane columns are the most common in liquid chromatography, as they allow the analysis and separation of many compounds, which turns out to be beneficial in the simultaneous analysis of multiple analytes, as was the case in the studies herein discussed [17,52].

Different types of detectors (UV/visible, MS, infrared, fluorescence, or electrochemical) can be used in liquid chromatography [52]. Different authors state that spectrophotometric methods are the most widely used in HPLC analysis, which was in line with the results since spectrophotometry is used in 70% of studies. [15,16,52].

**Figure 5:**
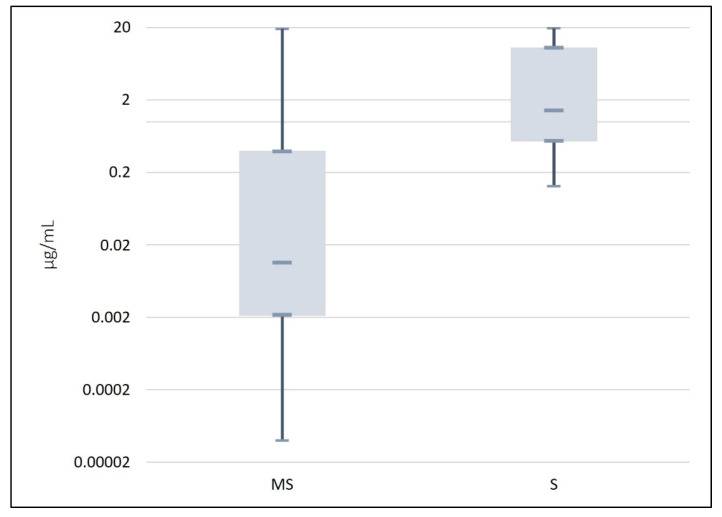
Box and whisker diagram of the limit of detection obtained in each study on chromatographic methods for quercetin quantification, published between 2018 and 2022, taking into consideration the detection method applied. MS: mass spectrometry; S: spectrophotometry.

For compounds that are not volatile or are not suitable for GC, liquid chromatography is normally used, usually coupled with DAD, which allows the analysis of thermolabile and non-volatile compounds [16,52,57,78,79,80]. In addition to the recognized greater sensitivity featured by MS in comparison with spectrophotometric methods, detection by MS also allows a better understanding of flavonoid structures [57]. The extent of greater sensitivity of MS as compared with spectrophotometric methods was depicted in Figure 5, where it can be observed a median LOD is about 100 times lower.

It was observed that 75% of the articles showed LOD values under 0.39 µg/mL when MS was used as the detection method, whereas when spectrophotometric methods were employed, 75% of the results were lower than 10.36 µg/mL. A comparison of the minimum values of the two methods showed a value of 0.00004 µg/mL in the MS detection method, whereas regarding the spectrophotometric method, a minimum value of 0.13 µg/mL was required for detection.

### 3.4. Validation Parameters

To guarantee the reliability of the results obtained and the reproducibility of the method, all analytical methods must undergo a validation process after the analysis of certain compounds [81,82,83].

Different parameters should be evaluated to ensure methods are properly validated. Among these parameters, the most important to guarantee the reproducibility of the method are sensitivity, precision, and accuracy [19,81,82,83,84].

Regarding sensitivity, this is given by the LOD (lowest concentration of analyte that the method can detect) and the LOQ (lowest concentration of analyte that the method can quantify) [19,81,82,83,84].

The methods reported in the 17 studies analyzed have shown very variable sensitivity values, ranging over different orders of magnitude.

For the agreement of successive measurements of the same method, carried out under the same conditions, expressed by the intra-day precision (also called repeatibility), the obtained values ranged from 92.2% to 99.73%. Regarding the degree of agreement between measurements made after promoting variations of different factors such as different days, different analysts, or different equipment, which is expressed by inter-day precision (also known as intermediate precision), the values reported in the analyzed studies ranged from 92.1% to 99.47%.

The guidelines state that the RSD must be less than 15% for each standard concentration tested, meaning that the precision must be greater than 85%. This aspect is verified in all the studies analyzed, which indicates that the methods reported have good repeatability and intermediate precision, which allows us to conclude that the methods developed make it possible to carry out precise quantification, even if small variations may occur [19,82,83].

In the included studies, the accuracy values were between 85.9% and 115%. Different methods can be used to determine this validation parameter. Among the different methods, the most commonly used are the use of reference materials, recovery tests, method comparison (comparing the results obtained with a reference method), and the standard addition method [19,81,82,83]. It was observed that the majority of studies used the recovery assessment method to determine accuracy [23,24,25,26,27,28,29,30,31,32,33,34,35,36,37,38,39]. This method consists of determining the percentage recovery in samples spiked with known concentrations of the analyte, being the most used technique in accuracy assessment [81,82].

### 3.5. Bias Assessment

For the assessment of bias related to the studies included in this review, as previously mentioned, the analysis of the studies was carried out based on eight criteria defined by Johnson [20].

After analyzing the studies according to the criteria defined for their evaluation, most of the studies addressed the same bias evaluation parameters defined by the Johnson [20] criteria, and all the included studies mentioned/presented, totally or partially, at least four of the eight parameters.

One of the parameters is the consideration of the difference plot and the statistics of the differences. In other words, a comparison should be made of the results obtained with the calibration line and its equation in samples of known concentration, with a subsequent comparison of the values and statistical presentation of the difference [20]. The difference plots and difference statistics were not addressed in any of the studies. The usually considered guidelines to perform the method’s validation do not mention the need to consider the difference plot and statistics of difference [19,85], but the disagreement in linear analysis is easily seen in difference plots (in which differences between the comparison estimates are plotted against the mean of their values), are hard to detect in x–y plots, that might wrongly suggest agreement [20,86].

Another parameter is the performance and interpretation of the interference test, which involves constructing two straight-line equations: one with just the standard in the injection solvent and another equation with the standard extracted into the matrix and then evaluating the difference [20]. Despite the importance of assessing the potential contribution of any interferent in an analytical method, the interference test was rarely presented [37,39]. Interferents can be endogenous (e.g., coming from matrix components) or exogenous (e.g., resulting from the carryover effect) [87].

To validate the results, the acceptable performance parameters (which results are acceptable or not) should be mentioned in the text before they are presented (introduction and/or methods), making this another criterion [20]. Only four studies unequivocally identified the criteria of acceptable performance to analyze and interpret the performance of the validation [35,37,38,39].

The evaluation of these studies also includes the presentation of the x–y plot of data with eye examination [20]. The presentation of the x–y plot of data in the published version of the study was only made in the study of Jan et al. [39] and supplementary data from four studies [23,28,31,33]. All the other studies also mentioned the construction of calibration curves and the subsequent x–y plot of data but did not allow ocular inspection by readers.

Another criterion is the performance and interpretation of the linearity test, in which the different parameters must be evaluated to ensure that the regression equation shows linearity within the defined range [20]. As to the linearity test, most studies interpreted the correlation coefficient obtained in the calibration curve to conclude about the linearity of the developed model and the suitability of the obtained equation [23,25,26,29,30,32,34,35,36,38,39]. Nonetheless, some authors consider that the correlation coefficient is not the most appropriate parameter for linearity determination and recommend that other evaluations should be performed, such as a relative error of the curve of less than 5% and zero contained in the ordinate of the origin [19,82,83,88].

Regression analysis is another of the parameters defined by Johnson [20] for evaluating articles on analytical techniques. In this case, all the studies presented the regression parameters (regression equation and coefficient of determination). These values make it possible to correctly analyze the adjustment of the curve and evaluate the results obtained in samples [81,82,83].

Regarding the last parameter, carrying out and interpreting the recovery test, the samples must be spiked, and the accuracy assessed according to the recovery method [20]. Only two studies did not assess recovery [36,37]. The guidelines generally used to validate analytical methods mention that accuracy can be determined using different methods and do not mention the need to carry out a recovery test [19,85].

Table 3 and Table 4 provide a general and specific analysis of the bias assessment for each parameter and by each study.

### 3.6. Assessment of the Methods

One of the main advantages of methods dedicated to a single analyte is the possibility of optimizing the method for detection with greater efficiency and lower solvent consumption. In the case of chromatographic analyses, efficiency is mainly reflected in retention time. Only one of the studies was exclusively dedicated to the detection of QUE but had a retention time of 32.9 min, much longer than other studies, ultimately resulting in higher solvent consumption [37]. For this reason, even if the intention is only to detect QUE, this study does not appear to be the most convenient option. The selection of any other method described in this review will, naturally, depend on the other compounds to be identified in addition to QUE. However, assuming the detection of QUE as the main objective, MS or spectrophotometry methods with retention times of less than 10 min combined with relatively low detection limits can be identified. If a quadrupole time-of-flight (QTOF) coupled to MS equipment is available, the method described by Sharma et al. [33] can be an interesting starting point for method development and/or adaptation, given the fact that it was the most sensible described method (LOD of 0.4 ng/mL) and presented a retention time of around 6 min. Furthermore, none of the MS methods completely satisfied more bias assessment parameters. Admitting that not all the laboratories possess MS equipment, the UHPLC-PDA method described by Srivastava et al. [27] might represent an alternative given the fact that it was the most sensitive spectrophotometric method with a retention time inferior to 10 min (LOD = 0.33 µg/mL; retention time = 6.4 min). However, if a higher content of QUE is to be expected and such a sensitive spectrophotometric method is not required, the method presented by Ramaswamy et al. [35] poses the best option. In this case, the retention time of QUE was 2.8 min, the LOD was 10 µg/mL, and a better performance in the bias assessment tool was obtained. Notably, the only study that performed better in the bias assessment carried out than the one by Ramaswamy et al. [35] was the one by Jan et al. [39]. It is also important to note that in the study by Ramaswamy et al. [35], an isocratic method is described, therefore providing a simpler method that prevents faster column degradation.

### 3.7. Limitations

The present review presents cutting-edge data on chromatographic analyses of QUE extracted from natural products, searchable through the PubMed search engine, that, unlike other databases, are free and universally accessible [89]. However, it is important to acknowledge that the included studies are limited owing to the use of a single database for their search, further increasing the publication bias. By searching other databases, such as Scopus and Web of Science, and extending the considered five-year period for search, it would be possible to access other relevant studies. Additionally, the inexistence of robust, validated, and generally accepted tools for bias assessment in analytical studies, as the ones included, hindered the performed bias assessment.

## 4. Conclusions

Several analytical methodologies for QUE detection from different sources in recent years have been developed. This fact further evidences the rising interest in QUE for different therapeutic purposes, which is also due to its availability in nature and its biological activities and traditional uses of plants containing it. Samples from plants have been depicted regarding their content in QUE, and although spectrophotometry methods were the most frequently validated, the present review enables the analysis of both MS and spectrophotometry methods. In addition to including studies that were validated regarding sensitivity, precision, and accuracy, it was concluded that all studies totally or partially accomplished at least half of the considered bias assessment criteria. This review provides an overview of validated methods, facilitating the adaptation of the most convenient method for those wishing to analyze QUE content according to their resources.

## Figures and Tables

**Table 1:** Summary of studies published between 2018 and 2022 describing chromatographic methods for the quantification of quercetin in plant sources regarding sample information, sample preparation, and amount of quercetin in real samples.

Reference	Analyte	Sample	Source	Sample Preparation and Extraction Procedures	Amount of Quercetin in Real Samples (µg/g)
Du et al. [23]	Chlorogenic acid; Cryptochlorogenic acid; Neochlorogenic acid; Isochlorogenic acid A; Isochlorogenic acid B; Isochlorogenic acid C; Caffeic acid; Hyperin; Isoquercitrin; Quercetin; Campherol; p-coumaric acid; Isorhamnetin; Rutin; Astragalin; Apigenin;	*Cuscuta chinensis* Lam.	Undisclosed	Pulverization; Ultra-sonication assisted extraction; Filtration (0.22 µm);	0.0735 ± 0.0788
Rajauria [24]	Phloroglucinol; Gallic acid; Cyanidin 3-glucoside; Chlorogenic acid; Rutin; Quercetin;	*Himanthalia* *elongata*	Seaweed	Grinding; Percolation; Solid-phase extraction; Filtration (0.22 µm);	4.2 ± 0.15
Yang et al. [25]	Alpinetin; Apigenin-7-O-β-D-glucopyranoside; Quercetin-3-O-β-D-glucopyranoside; Scutellarein; Apigenin; Wogonoside; Quercetin; Amentoflavone; Wogonin; Chrysin; Luteolin; Rutin; Naringenin; Baicalein; Baicalin;	*Scutellaria barbata* D. Don and *Hedyotis* *diffusa* (Willd.) Roxb.	Dry Grass (Plants)	Reflux extraction (twice); Lyophilization; Solvent resuspension; Liquid–liquid extraction; Filtration (0.22 µm);	0.02199 ± 0.000618
Zhou et al. [26]	Myricetin-3-O-β-D-galactoside; Myricetin-3-O-glucoside; Quercetin3-O-β-D-galactoside; Quercetin-3-O-β-D-glucoside; Quercetin-3-O-(2″-O-galloyl-β-d-galactoside); Quercetin-3-O(2″-O-galloyl-β-d-glucoside); Kaempferol-3-O-β-D-galactoside; Kaempferol-3-O-β-D-glucoside; Kaempferol-3-O(2″-O-galloyl-β-D-galactoside); Kaempferol-3-O-(2″-O-galloyl-β-D-glucoside); Quercetin; Kaempferol;	*Diospyros khaki*	Leaves (Plant)	Grinding; Reflux extraction (twice); Defat procedure (twice); Liquid–liquid extraction (twice); Gel Column Chromatography;	12,700 ± 8000
Srivastava et al. [27]	Acteoside; Isoacteoside; Durantoside-I; Quercetin; Methylapigenin-7-O-D-glucopyranuronate;	*Duranta erecta* L.	Undisclosed	Pulverization; Ultra-sonication assisted extraction; Filtration (0.22 µm);	2010
Pu et al. [28]	Hydroxysafflor yellow A; Safflomin C; Anhydrosafflor yellow B; Kaempferol; Kaempferol-3-O-glucoside; Kaempferol-3-O-rutinoside; Kaempferol-3-O-β-sophoroside; 6-hydroxykaempferol; 6-hydroxykaempferol-3-O-β-D-glucoside; 6-hydroxykaempferol-3,6-di-O-β-D-glucoside; 6-hydroxykaempferol-3,6,7-tri-O-β-D-glucoside; Quercetin; Rutin; Luteoloside; Apigenin; Quercetin-3-O-β-D-glucoside;	*Carthamus* *tinctorius* L.	Undisclosed	Pulverization; Ultra-sonication assisted extraction; Filtration (0.22 µm);	65 ± 75
Huang et al. [29]	Chlorogenic acid; Rutin; Isoquercetrin; Nictoflorin; Astragalin; Quercetin;	*Sambucus* *formosana*	Stems, leaves, and roots (Plant)	Pulverization; Percolation; Liquid–liquid extraction (twice);	3500 ± 70
Chen et al. [30]	Gallic acid; Chlorogenic acid; Caffeic acid; Syringic acid; p-coumaric acid; Ferulic acid; Benzoic acid; Salicylic acid; Catechin; Epicatechin; Rutin; Naringin; Hesperidin; Quercetin; Resveratrol; Nobiletin; Tangeritin;	Chinese citrus and grape	Fruit (Plant)	Percolation; Liquid–liquid extraction (twice); Filtration (0.45 µm);	394,800 ± 527,900 (citrus) 129,700 ± 146,600 (grape)
Khan et al. [31]	6‴-feruloylspinosin; Apigenin; Apigenin-7-O-glucoside; Catechin; Jujuboside A; Jujuboside B; Luteolin; Quercetin;	*Ziziphus* *jujuba* and *Ziziphus* *nummularia*	Fruits (Plants)	Grinding; Ultra-sonication assisted extraction; Filtration 0.22 µm;	15.5 ± 12.0
Jia et al. [32]	Phloretin; Gallic acid; Protocatechuat E; Catechin; 2,4-dihydroxybenzoic acid; Chlorogenic acid; Proanthocyanidins-B2; Vanillic acid; O-hydroxybenzene acetic acid; Coffeic acid; Syringate; p-coumaric acid; Proanthocyanidins-A2; Veratronic acid; Ferulic acid; Benzoic acid; Salicylic acid; Naringin; Hesperidin; Rutin; Ellagic acid; Myricetin; Naringenin; Quercetin; Kaempferol;	Berries	Fruit (Plant)	Grinding; Ultra-sonication assisted extraction; Filtration; Lyophilization; Solvent resuspension; Filtration (0.22 µm);	11.5 ± 15.5
Sharma et al. [33]	Rutin; Quercetin; Kaempherol; 5,7-dihydroxy-3-(2-hydroxy-4-methoxybenzyl)chroman-4-one; 5,7-dihydroxy-3-(2-hydroxy-4-methoxybenzyl)8-methylchroman-4-one; 5,7-dihydroxy-3-(4-methoxybenzyl)8-methylchroman-4-one;	*Polygonatum* *verticillatum*	Rhizomes (Plant)	Pulverization; Percolation (fivefold); Liquid–liquid extraction; Filtration (0.25 µm);	0.0243 ± 0.0044
Sharma et al. [34]	Quercetin; Ferulic acid; Chlorogenic acid;	*Myristic* *fragrans,* *Hemidesmus* *indicus,* *and* *Inula* *racemosa*	Undisclosed	Maceration; Filtration (11 µm); Lyophilization; Solvent resuspension; Filtration (undisclosed diameter);	0.0062
Ramaswamy et al. [35]	Curcumin; Piperine; Quercetin; Rutin;	*Camellia sinensis* L. (1); *Glycyrrhiza glabra* L. (2); *Thymus* *vulgaris* L. (3); *Citrus* *aurantium* L. (4);	Leaves (1, 3), rhizomes (2), tuberous roots (2), and rind (4) (Plants)	Ultra-sonication assisted extraction; Filtration 0.22 µm;	*C. s*: 0.0036 *C. a*: 0.0011 *G. g*: 0.00095 *T. v*: 0.00087
Ali et al. [36]	Rutin; Taxifolin; Quercetin; Apigenin; Kaempferol; Betulinic acid; Oleanolic acid; Betulin; Lupeol; Stigmasterol; β-sitosterol; Ursolic acid;	*Caesalpinia* *pulcherrima* (1); *Citrus lemon* (2); *Opuntia* *dellenii* (3); *Bauhinia* *variegata* (4); *Polyalthia longifolia* var. pendula (5); *Bombax ceiba* (6); *Phlox drummondii* (7); *Olea europea* (8); *Tagetes* *patula* (9); *Melia* *azedarach* (10);	Flower (1, 9, 10), fresh pods (1), seeds (2), cladodes (3), pod (4), root bark (5), wood (6), aerial part (7), leaves (8), and stem bark (6) (Plants)	Ultra-sonication assisted extraction; Filtration 0.22 µm;	*C. p* (flowers): 234.56 µg/mL *C. p* (fresh pods): 315.07 µg/mL *C. l*: < LOQ *O. d*: < LOQ *B. v*: < LOQ *P. l*: 579.51 µg/mL *B. c*: < LOQ *P. d*: < LOQ *O. e*: 94.50 µg/mL *T. p*: < LOQ
Macêdo et al. [37]	Quercetin	*Triplaris* *gardneriana* Wedd	Leaves (Plant)	Pulverization; Percolation (threefold); Vacuum Liquid Chromatography;	9967 ± 1010
Urbstaite et al. [38]	Chlorogenic acid; Myricetin-3-galactoside; Quercetin-3-galactoside; Quercetin-3-glucoside; Quercetin-3-α-Larabinopyranoside; Quercetin-3-α-L-arabinofuranoside; Quercetin-3-rhamnoside; Myricetin; Quercetin;	*Vaccinium* *macrocarpon* Aiton	Fruit (Plant)	Pulverization; Ultra-sonication assisted extraction; Filtration (0.22 µm);	89.76 ± 1.58
Jan et al. [39]	Rutin and Quercetin	Buckwheat (*Fagopyrum* spp.)	Seeds and Leaves (Plant)	Pulverization; Percolation; Filtration (0.22 µm);	0.00011 ± 0.00014

*B. c*: *Bombax ceiba*; *B. v*: *Bauhinia variegata*; *C. a*: *Citrus aurantium* L.; *C. l*: *Citrus lemon*; *C. s*: *Camellia sinensis* L.; *C. p*: *Caesalpinia pulcherrima*; *G. g*: *Glycyrrhiza glabra* L.; *O. d*: *Opuntia dellenii*; *O. e*: *Olea europea*; *P. d*: *Phlox drummondii*; *P. l*: *Polyalthia longifolia*; *T. p*: *Tagetes patula*; *T. v*: *Thymus vulgaris* L.

**Table 2:** Summary of studies published between 2018 and 2022 describing chromatographic methods for the quantification of quercetin in plant sources regarding chromatographic and detection conditions and validation parameters.

Reference	Analytical Method						Validation Parameters			
	Chromatographic Method	Detection Method	Chromatographic Run	Mobile Phase	Column	Retention Time (min)	LOD (µg/mL)	LOQ (µg/mL)	Precision (%)	Accuracy (%)
Du et al. [23]	HPLC	ESI-MS	Gradient	acetonitrile + water acidified with 0.05% formic acid	C18 (1.8 μm, 4.6 mm × 150 mm)	17.25	0.03	0.1	Intra-day: 92.2–95.4 Inter-day: 92.1–99.0	Intra-day: 102.3–110.3 Inter-day: 107.0–115.0
Rajauria [24]	RP-HPLC	DAD-ESI-MS	Gradient	0.25% aqueous acetic acid and acetonitrile/water (80/20; *v*/*v*) containing 0.25% acetic acid	C-18 (5 μm, 4.6 mm × 250 mm)	37.43	0.51	1.82	Retention Time: 98.17 Peak Area: 96.37	Recovery: 97.2
Yang et al. [25]	HPLC	Q-TOF-MS	Gradient	water containing 0.1% formic acid and acetonitrile containing 0.1% formic acid	C18 (5 μm, 4.6 mm × 150 mm)	7.07	0.003	0.0105	Intra-day: 99.28 Inter-day: 97.65	Recovery: 96.0–103.0
Zhou et al. [26]	HPLC	DAD-Q-TOF-MS/MS DAD	Gradient Isocratic	acetonitrile and water containing 0.1% formic acid	C18 (5 μm, 2.1 mm × 150 mm, 100 A)	32.11	0.015	0.051	Intra-day: 97.2–99.4 Inter-day: 97.0–99.2	Recovery: 85.9–106.9
Srivastava et al. [27]	UHPLC	PDA	Gradient	water containing 0.1% formic acid and acetonitrile	C18 (2.5 μm, 2.0 mm × 100 mm)	6.4	0.330	1.101	Intra-day: 99.06 Inter-day: 97.64–98.16	Recovery: 101.0
Pu et al. [28]	UPLC	QTRAP^®^-MS2	Gradient	0.1% formic acid aqueous solution and acetonitrile	C18 (1.7 μm, 2.1 mm × 100 mm)	12.56	0.007629	0.015259	Intra-day: 96.06 Inter-day: 97.12	Recovery: 98.67–103.55
Huang et al. [29]	HPLC	DAD-ESI-MS	Gradient	0.1% formic acid aqueous solution and 0.1% formic acid/acetonitrile	RP-C18 (1.9 μm, 3 mm × 100 mm)	18.9	0.8	2.5	97.3	Recovery: 92.7
Chen et al. [30]	HPLC	DAD	Gradient	water with 2% (*v*/*v*) acetic acid and acetonitrile	RP-18e (5 μm, 4.0 mm × 250 mm)	76.52	0.13	0.39	Repeatability: 98.03	Recovery: 94.74
Khan et al. [31]	HPLC	ESI-Q-TOF-MS	Gradient	water with 0.1% formic acid and methanol with 0.1% formic acid	SB-C18 (1.8 μm, 3.0 mm × 50 mm)	4.9	0.00028	0.00086	Intra-day: 96.2–98.3 Inter-day: 97.4–98.5	Recovery: 98.3–101.4
Jia et al. [32]	UPLC	Q-Orbitrap MS	Gradient	water containing 0.1% formic acid and 0.1% of formic acid in methanol	C18 (2.6 μm 2.1 mm × 150 mm)	16.31	0.00187	0.00695	Intra-day: 98.41 Inter-day: 97.77	Recovery: 96.2–99.2
Sharma et al. [33]	UHPLC	DAD-Q-TOF-MS	Gradient	water and acetonitrile, containing 0.1% formic acid	C18 (1.8 μm, 2.1 mm × 150 mm)	5–6	0.00004	0.00012	Intra-day: 98.29 Inter-day: 97.74	Recovery: 93.5
Sharma et al. [34]	RP-HPLC	UV-Vis	Isocratic	Acetonitrile and 0.1 M orthophosphoric acid in water with pH 2.5 in a ratio of 75 + 25 (*v*/*v*)	N/A	7.44	1.41	6.54	>98	Recovery: 94.65–98.14
Ramaswamy et al. [35]	UFLC	PDA	Isocratic	Ammonium acetate buffer (25 mM, pH 3.0) and acetonitrile (20:80, *v*/*v*)	C18 (5 μm, 4.6 mm × 250 mm)	2.8	10	30	Intra-day: 98.49–99.01 Inter-day: 98.22–99.31	Recovery: 98.88
Ali et al. [36]	HPLC	DAD/ESI-MS/MS	Gradient	water plus 0.1% formic acid and acetonitrile with 0.1% formic acid	C18 (1.8 μm, 3 mm × 100 mm)	8.10	19.1	57.9	Intra-day: 92.79–99.5 Inter-day: 98.78–99.47	Intra-day: 104.59–119.95 Inter-day: 100.91–115.64
Macêdo et al. [37]	HPLC	DAD	Gradient	water containing 0,3% formic acid and methanol	RP C-18 (5 μm, 4.6 mm × 250 mm)	32.9	10.72	35.75	Intra-day: 96.34–99.73 Inter-day: 94.62–98.71	94.83–100.84
Urbstaite et al. [38]	UPLC	PDA	Gradient	0.1% formic acid (*v*/*v*) in water and acetonitrile	C18 (1.7 μm, 2.1 mm × 100 mm)	12.104	0.76	2.29	Intra-day: 98.7 Inter-day: 98.24	Recovery: 97.12–101.19
Jan et al. [39]	HPLC	DAD	Gradient	methanol and methanol:water:acetic acid in the ratio of 100:150:5	C18 (5 μm, 4.6 mm × 150 mm)	8.23	19.28	1.77	Intra-day: 98.75 Inter-day: 97.27	Recovery: 96.66–98.63

DAD: diode array detector; ESI: electrospray ionization; HPLC: high-performance liquid chromatography; LOD: limit of detection; LOQ: limit of quantification; MS: mass spectrometry; PDA: photodiode array detector; Q-TOF: quadrupole time-of-flight; Q-TRAP: quadrupole ion trap; RP: reverse-phase; UFLC: ultra-fast liquid chromatography; UPLC: ultra-performance liquid chromatography; UHPLC: ultra-high-performance liquid chromatography; UV-vis: ultraviolet-visible.

**Table 3:** Identification of bias assessment parameters and general overview of fulfillment.

Bias Assessment Parameter Code	Explanation	Accomplishing the Parameter (n (%))
I	Establishment of criteria for acceptable performance	4 (24%)
II	Comparison of test method with reference method using reference material	17 (100%)
III	Presentation of the x–y plot of data with an eye examination	17 (100%)
IV	Consideration of difference plots and statistics of difference	0 (0%)
V	Consideration of regression analysis	17 (100%)
VI	Performance and interpretation of interference test	2 (12%)
VII	Performance and interpretation of linearity test	13 (76%)
VIII	Performance and interpretation of recovery test	15 (88%)

**Table 4:** Qualitative evaluation of the studies, based on the parameters defined for assessing bias (red color = not mentioned/presented; green color = mentioned/presented; yellow color = partially mentioned/presented).

Reference	Bias Assessment Parameters							
	I	II	III	IV	V	VI	VII	VIII
Du et al. [23]							b	
Rajauria [24]			a					
Yang et al. [25]			a				b	
Zhou et al. [26]			a				b	
Srivastava et al. [27]			a					
Pu et al. [28]								
Huang et al. [29]			a				b	
Chen et al. [30]			a				b	
Khan et al. [31]								
Jia et al. [32]			a				b	
Sharma et al. [33]								
Sharma et al. [34]			a				b	
Ramaswamy et al. [35]			a				b	
Ali et al. [36]			a				b	
Macêdo et al. [37]			a					
Urbstaite et al. [38]			a				b	
Jan et al. [39]							b	

a The calibration curve and respective x–y plot of data were mentioned but not graphically exhibited. b Interpreted accordingly to the correlation coefficient.

## Data Availability

Data are contained within the article and supplementary materials.

## Supplementary Materials

The following supporting information can be downloaded at https://www.mdpi.com/article/10.3390/molecules28237714/s1, Table S1: Time programs for gradient separation of studies published between 2018 and 2022 describing chromatographic methods for the quantification of quercetin in plant sources. Table S2: PRISMA checklist for Systematic Reviews adapted from Page et al. [90]; Table S3: PRISMA checklist for abstracts for Systematic Reviews adapted from Page et al. [90].
