# Supplementary material for: Chromatographic Methods Developed for the Quantification of Quercetin Extracted from Natural Sources: Systematic Review of Published Studies from 2018 to 2022
Source: Molecules. 2023 Nov 22;28(23):7714. doi: 10.3390/molecules28237714 (PMC10708206; doi:10.3390/molecules28237714)
Supplement: Supplementary file 1 [file molecules-28-07714-s001.zip › Table S1.pdf]

Table S1. Gradient program employed in papers published between 2018 and 2022 describing chromatographic methods for the quantification of quercetin in plant sources.

Reference	Gradient Program
Du *et al.* [1]	15% to 19% acetonitrile and 85% to 81% 0.05% aqueous formic acid in water at 0–3 min, 19% to 20% acetonitrile and 81% to 80% 0.05% aqueous formic acid in water at 3–9 min, 20% to 30% acetonitrile and 80% to 70% 0.05% aqueous formic acid in water at 9–12 min, 30% to 48% acetonitrile and 70% to 52% 0.05% aqueous formic acid in water at 12–12.5 min, 48% to 52% acetonitrile and 52% to 48% 0.05% aqueous formic acid in water at 12.5-14 min, 52% to 54% acetonitrile and 48% to 46% 0.05% aqueous formic acid in water at 14–17 min, 54% to 60% acetonitrile and 46% to 40% 0.05% aqueous formic acid in water at 17–18.5 min, and then 60% to 81% acetonitrile and 40% to 19% 0.05% aqueous formic acid in water at 18.5-20 min
Rajauria [2]	90% 0.25% aqueous acetic acid and 10% acetonitrile/water (80/20 v/v) isocratic at 0-20 min, 20–30 min linear gradient from 90% to 80% of 0.25% aqueous acetic acid and 10% to 20% of acetonitrile/water (80/20 v/v), 30–35 min linear gradient from 80% to 70% of 0.25% aqueous acetic acid and 20% to 30% acetonitrile/water (80/20 v/v), 35–45 min 100% 0.25% aqueous acetic acid and 0% acetonitrile/water (80/20 v/v) isocratic
Yang *et al.* [3]	65% to 30% water containing 0.1% formic acid and 35% to 70% acetonitrile containing 0.1% formic acid in 0–15 min, 30% water containing 0.1% formic acid and 70% acetonitrile containing 0.1% formic acid in 16–17 min, 5% water containing 0.1% formic acid and 95% acetonitrile containing 0.1% formic acid for 18–20 min, and 5% to 65% water containing 0.1% formic acid and 95% to 35% acetonitrile containing 0.1% formic acid for 21–25 min
Zhou *et al.* [4]	14% to 18% acetonitrile and 86% to 82% water containing 0.1% formic acid at 0–10 min, 18% to 20% acetonitrile and 82% to 80% water containing 0.1% formic acid at 10–25 min, and 20% to 30% acetonitrile and 80% to 70% water containing 0.1% formic acid at 25–45 min
Srivastava *et al.* [5]	95% to 78% water containing 0.1% formic acid and 5% to 22% acetonitrile at 0–1 min, 78% to 70% water containing 0.1% formic acid and 22% to 30% acetonitrile at 1–6.50 min, 70% to 60% water containing 0.1% formic acid and 30% to 40% acetonitrile at 6.50–7 min, 60% water containing 0.1% formic acid and 40% acetonitrile at 7–10 min, and increasing from 60% to 95% water containing 0.1% formic acid and decreasing from 40% to 5% acetonitrile at 10–11 min
Pu *et al.* [6]	90% to 80% 0.1% formic acid aqueous solution and 10% to 20% acetonitrile at 0–2 min, 80% to 70% 0.1% formic acid aqueous solution and 20% to 30% acetonitrile at 2–7 min, 70% to 20% 0.1% formic acid aqueous solution and 30% to 80% acetonitrile at 7–12 min, 20% to 0% 0.1% formic acid aqueous solution and 80% to 100% acetonitrile at 12–13 min, and 0% to 90% 0.1% formic acid aqueous solution and 100% to 10% acetonitrile at 13–14 min
Huang *et al.* [7]	85% 0.1% formic acid aqueous solution and 15% 0.1% formic acid/acetonitrile from 0 to 5 min, 65% to 45% 0.1% formic acid aqueous solution and 35% to 55% 0.1% formic acid/acetonitrile during 3 to 8 min, 45% to 0% 0.1% formic acid aqueous solution and 55% to 100% 0.1% formic acid/acetonitrile during 8 to 15 min
Chen *et al.* [8]	96% water with 2% acetic acid and 4% acetonitrile in the first 5 min, 96% to 92% water with 2% acetic acid and 4% to 8% acetonitrile at 18 min; kept isocratically with 92% water with 2% acetic acid and 8% acetonitrile for the following 22 min, then decreased to 70% water with 2% acetic acid and increased to 30% acetonitrile at 75 min, 50% water with 2% acetic acid and 50% acetonitrile within next 15 min followed by another isocratic step with 50% water with 2% acetic acid and 50% acetonitrile for 5 min, and linear gradient back to 96% water with 2% acetic acid and 4% acetonitrile in 5 min

Khan *et al.* [9]	80% water with 0.1% formic acid and 20% methanol with 0.1% formic acid, and then raised to 30% water with 0.1% formic acid and 70% methanol with 0.1% formic acid in 1 min which was then kept constant for 1.0 min, and further raised to 5% water with 0.1% formic acid and 95% methanol with 0.1% formic acid in 1.5 min, lowered back to 80% water with 0.1% formic acid and 20% methanol with 0.1% formic acid in 1.0 min and then 1.0 min of equilibration time was given
Jia *et al.* [10]	98% to 35% water containing 0.1% formic acid and 2-65% 0.1% of formic acid in methanol at 0-18 min, 35% to 5% water containing 0.1% formic acid and 65-95% 0.1% of formic acid in methanol at 18-20 min, 5% water containing 0.1% formic acid and 95% 0.1% of formic acid in methanol at 20-21 min, 5% to 98% water containing 0.1% formic acid and 95-2% 0.1% of formic acid in methanol at 21-23 min, 98% water containing 0.1% formic acid and 2% 0.1% of formic acid in methanol at 23-25 min
Sharma *et al.* [11]	90% water containing 0.1% formic acid and 10% acetonitrile at 0-4 min, 90% water containing 0.1% formic acid and 10% acetonitrile at 4-70 min, 10% water containing 0.1% formic acid and 90% acetonitrile at 9 min, and a final step was re-equilibration washing with 90% water containing 0.1% formic acid and 10% acetonitrile for 2 min
Sharma *et al.* [12]	N/A
Ramaswamy *et al.* [13]	N/A
Ali *et al.* [14]	80% water plus 0.1% formic acid and 20% acetonitrile with 0.1% formic acid at 0–2 min, 80% to 55% water plus 0.1% formic acid and 20% to 45% acetonitrile with 0.1% formic acid at 2–10 min, 55% to 15% water plus 0.1% formic acid and 45% to 85% acetonitrile with 0.1% formic acid at 10–11 min, 15% to 12% water plus 0.1% formic acid and 85% to 88% acetonitrile with 0.1% formic acid at 11–17 min, 12% to 0% water plus 0.1% formic acid and 88% to 100% acetonitrile with 0.1% formic acid at 17–18 min, 0% water plus 0.1% formic acid and 100% acetonitrile with 0.1% formic acid at 18–25 min, 0% to 80% water plus 0.1% formic acid and 100–20% acetonitrile with 0.1% formic acid at 25–27 min, 80% water plus 0.1% formic acid and 20% acetonitrile with 0.1% formic acid at 27–29 min
Macêdo *et al.* [15]	80% water containing 0,3% formic acid and 20% methanol at 0 min, 80% water containing 0,3% formic acid and 20% methanol at 5 min, 65% water containing 0,3% formic acid and 35% methanol at 20 min, 50% water containing 0,3% formic acid and 50% methanol at 25 min, 30% water containing 0,3% formic acid and 70% methanol at 28 min, 30% water containing 0,3% formic acid and 70% methanol at 35 min, 80% water containing 0,3% formic acid and 20% methanol at 37 min, and 80% water containing 0,3% formic acid and 20% methanol at 45 min
Urbstaite *et al.* [16]	95% water containing 0.1% formic acid and 5% acetonitrile at 0 min, 88% water containing 0.1% formic acid and 12% acetonitrile at 1 min, 88% water containing 0.1% formic acid and 12% acetonitrile at 3 min, 87% water containing 0.1% formic acid and 13% acetonitrile at 4 min, 75% water containing 0.1% formic acid and 25% acetonitrile at 9 min, 70% water containing 0.1% formic acid and 30% acetonitrile at 10.5 min, 70% water containing 0.1% formic acid and 30% acetonitrile at 12 min, 10% water containing 0.1% formic acid and 90% acetonitrile at 12.5 min, 10% water containing 0.1% formic acid and 90% acetonitrile at 13 min, 95% water containing 0.1% formic acid and 5% acetonitrile at 13.5 min, and 95% water containing 0.1% formic acid and 5% acetonitrile at 14.5 min, delaying the next injection by 2 min
Jan *et al.* [17]	0% methanol and 100% methanol:water:acetic acid (100:150:5) at 0 min, 0% methanol and 100% methanol:water:acetic acid (100:150:5) at 4 min, 0% methanol and 100% methanol:water:acetic acid (100:150:5) at 15 min

N/A: not applicable.

References

1. Du, K.; Li, J.; Guo, X.; Li, Y.; Chang, Y. Quantitative Analysis of Phenolic Acids and Flavonoids in *Cuscuta chinensis* Lam. by Synchronous Ultrasonic-Assisted Extraction with Response Surface Methodology. *J Anal Methods Chem* **2018**, *2018*, doi:10.1155/2018/6796720.
2. Rajauria, G. Optimization and Validation of Reverse Phase HPLC Method for Qualitative and Quantitative Assessment of Polyphenols in Seaweed. *J Pharm Biomed Anal* **2018**, *148*, 230–237, doi:10.1016/j.jpba.2017.10.002.
3. Yang, W.; He, S.; Xiao, N.; Qiao, Y.; Sui, H.; Liang, L.; Chen, J.; Li, W.; Zhang, L. Simultaneous Determination of 15 Flavonoids in *Scutellaria barbata-Hedyotis diffusa* Herb Pair by HPLC Q-TOF MS. *J AOAC Int* **2019**, *102*, 75–80, doi:10.5740/jaoacint.17-0469.
4. Zhou, Z.; Li, N.; Zhang, H.-F.; Wang, Q.-Q.; Yu, Q.; Wang, F.; Dai, Y.-H.; Wang, D.; Liu, D.-C. Simultaneous Quantitative Analysis of 11 Flavonoid Derivatives with a Single Marker in Persimmon Leaf Extraction and Evaluation of Their Myocardium Protection Activity. *J Nat Med* **2019**, *73*, 404–418, doi:10.1007/s11418-018-1274-y.
5. Srivastava, M.; Singh, M.; Maurya, P.; Srivastava, N.; Gupta, N.; Shanker, K. Simultaneous Quantification of Five Bioactive Phenylethanoid, Iridoid, and Flavonol Glycosides in *Duranta erecta* L.: Ultra Performance Liquid Chromatography Method Validation and Uncertainty Measurement. *J Pharm Biomed Anal* **2019**, *174*, 711–717, doi:10.1016/j.jpba.2019.06.044.
6. Pu, Z.-J.; Yue, S.-J.; Zhou, G.-S.; Yan, H.; Shi, X.-Q.; Zhu, Z.-H.; Huang, S.-L.; Peng, G.-P.; Chen, Y.-Y.; Bai, J.-Q.; *et al.* The Comprehensive Evaluation of Safflowers in Different Producing Areas by Combined Analysis of Color, Chemical Compounds, and Biological Activity. *Molecules* **2019**, *24*, 3381, doi:10.3390/molecules24183381.
7. Huang, H.-S.; Yu, H.-S.; Yen, C.-H.; Liaw, E.-T. HPLC-DAD-ESI-MS Analysis for Simultaneous Quantitation of Phenolics in Taiwan Elderberry and Its Anti-Glycation Activity. *Molecules* **2019**, *24*, 3861, doi:10.3390/molecules24213861.
8. Chen, Y.; Hong, Y.; Yang, D.; He, Z.; Lin, X.; Wang, G.; Yu, W. Simultaneous Determination of Phenolic Metabolites in Chinese Citrus and Grape Cultivars. *PeerJ* **2020**, *8*, e9083, doi:10.7717/peerj.9083.
9. Khan, M.N.; Ul Haq, F.; Rahman, S.; Ali, A.; Musharraf, S.G. Metabolite Distribution and Correlation Studies of *Ziziphus jujuba* and *Ziziphus nummularia* Using LC-ESI-MS/MS. *J Pharm Biomed Anal* **2020**, *178*, 112918, doi:10.1016/j.jpba.2019.112918.
10. Jia, Q.; Zhang, S.; Zhang, H.; Yang, X.; Cui, X.; Su, Z.; Hu, P. A Comparative Study on Polyphenolic Composition of Berries from the Tibetan Plateau by UPLC-Q-Orbitrap MS System. *Chem Biodivers* **2020**, *17*, e2000033, doi:10.1002/cbdv.202000033.
11. Sharma, S.; Joshi, R.; Kumar, D. Quantitative Analysis of Flavonols, Flavonol Glycoside and Homoisoflavonoids in *Polygonatum verticillatum* Using UHPLC-DAD-QTOF-IMS and Evaluation of Their Antioxidant Potential. *Phytochem Anal PCA* **2020**, *31*, 333–339, doi:10.1002/pca.2899.
12. Sharma, A.; Katiyar, C.K.; Banerjee, S.; Chanda, J.; Kar, A.; Biswas, S.; Mukherjee, P.K. RP-HPLC and HPTLC Methods for Analysis of Delected Herbs Used as Complexion Promoters in Ayurveda and Unani Systems of Medicine. *J AOAC Int* **2020**, *103*, 692–698, doi:10.5740/jaoacint.19-0290.
13. Ramaswamy, S.; Gowthamarajan, K.; Priyanka Dwarampudi, L.; Bhaskaran, M.; Kadiyala, M. Analytical Method Development, Validation and Forced Degradation Studies for Rutin, Quercetin, Curcumin, and Piperine by RP-UFLC Method. *Drug Dev Ind Pharm* **2021**, *47*, 562–568, doi:10.1080/03639045.2021.1892740.
14. Ali, K.; Ali, A.; Khan, M.N.; Rahman, S.; Faizi, S.; Ali, M.S.; Khalifa, S.A.M.; El-Seedi, H.R.; Musharraf, S.G. Rapid Identification of Common Secondary Metabolites of Medicinal Herbs Using High-Performance Liquid Chromatography with Evaporative Light Scattering Detector in Extracts. *Metabolites* **2021**, *11*, 489, doi:10.3390/metabo11080489.
15. Macêdo, S.K.S.; Almeida, T.S.; Alencar Filho, J.M.T.; Lima, K.S.B.; Libório, R.C.; Costa, M.M.; Rolim Neto, P.J.; Rolim, L.A.; Nunes, X.P. Phytochemical Identification and Quantification of Quercetin in *Triplaris gardneriana* Wedd. Leaves by HPLC-DAD with Evaluation of Antibacterial Activity. *Nat Prod Res* **2021**, *35*, 3083–3088, doi:10.1080/14786419.2019.1682573.
16. Urbstaite, R.; Raudone, L.; Liaudanskas, M.; Janulis, V. Development, Validation, and Application of the UPLC-DAD Methodology for the Evaluation of the Qualitative and Quantitative Composition of Phenolic Compounds in the Fruit of American Cranberry (*Vaccinium macrocarpon* Aiton). *Molecules* **2022**, *27*, 467, doi:10.3390/molecules27020467.

17. Jan, S.; Ahmad, J.; Dar, M.M.; Wani, A.A.; Tahir, I.; Kamili, A.N. Development and Validation of a Reverse Phase HPLC-DAD Method for Separation, Detection & Quantification of Rutin and Quercetin in Buckwheat (*Fagopyrum* spp.). *J Food Sci Technol* **2022**, *59*, 2875–2883, doi:10.1007/s13197-021-05312-0.
